# Long-term repeatability of cognitive performance

**DOI:** 10.1098/rsos.220069

**Published:** 2022-05-24

**Authors:** Benjamin J. Ashton, Alex Thornton, Maxime Cauchoix, Amanda R. Ridley

**Affiliations:** ^1^ School of Natural Sciences, Macquarie University, Sydney, New South Wales 2109, Australia; ^2^ School of Biological Sciences, University of Bristol, 24 Tyndall Avenue, Bristol BS8 1TQ, UK; ^3^ Centre for Evolutionary Biology, School of Biological Sciences, University of Western Australia, Perth, Western Australia 6009, Australia; ^4^ Centre for Ecology and Conservation, University of Exeter, Penryn Campus, Treliever Road, Penryn TR10 9FE, UK; ^5^ Station d'Ecologie Théorique et Expérimentale du CNRS (UMR5321), Moulis, France

**Keywords:** cognition, cognitive performance, repeatability, Australian magpie

## Abstract

Measures of cognitive performance, derived from psychometric tasks, have yielded important insights into the factors governing cognitive variation. However, concerns remain over the robustness of these measures, which may be susceptible to non-cognitive factors such as motivation and persistence. Efforts to quantify short-term repeatability of cognitive performance have gone some way to address this, but crucially the long-term repeatability of cognitive performance has been largely overlooked. Quantifying the long-term repeatability of cognitive performance provides the opportunity to determine the stability of cognitive phenotypes and the potential for selection to act on them. To this end, we quantified long-term repeatability of cognitive performance in wild Australian magpies over a three-year period. Cognitive performance was repeatable in two out of four cognitive tasks—associative learning and reversal-learning performance was repeatable, but spatial memory and inhibitory control performance, although trending toward significance, was not. Measures of general cognitive performance, obtained from principal components analyses carried out on each cognitive test battery, were highly repeatable. Together, these findings provide evidence that at least some cognitive phenotypes are stable, which in turn has important implications for our understanding of cognitive evolution.

## 1. Introduction

Studies investigating cognitive evolution have traditionally taken a comparative approach [1–3], but findings are contradictory and contentious [4]. More recently, an intraspecific approach has been championed, focusing on the causes and consequences of individual variation in cognitive performance on psychometric tasks [5]. Studies using this approach have identified that variation in the social environment [6,7], altitude [8], environmental unpredictability [9] and predation pressure [10] predict differences in cognitive performance between populations or individuals. In addition, a number of studies have identified relationships between cognitive performance and survival or reproductive success [6,11–13]. As such, the use of psychometric tasks, focusing on intraspecific variation in cognition, has been identified as a powerful approach to complement comparative and neuroanatomical approaches to the study of cognition [5].

To demonstrate that measures of cognitive performance derived from psychometric tasks are robust and consistent, it is crucial to quantify repeatability of performance. This is particularly important because performance on cognitive tasks can be influenced by non-cognitive confounding variables such as prior experience, motivation, persistence, or energetic state [14–16]. High repeatability of cognitive performance suggests that measures of performance are robust, as it indicates that performance is not determined by confounding variables that are likely to vary over short time-frames (e.g. energetic state). Furthermore, the biological sciences, and science at large, are in the midst of a replication crisis [17,18]—repeated testing of individuals will help to address this by determining whether cognitive performance at one point in time is indicative of cognitive performance at a later point, going some way to ensuring the validity of results.

The vast majority of studies that have quantified the repeatability of cognitive performance have done so across relatively short timeframes (see Cauchoix *et al*. [19] and references therein), typically in the range of days to weeks between tasks (although see Soha *et al.* [20], Davidson *et al.* [21], and Cole *et al.* [22] for notable exceptions). A recent meta-analysis of cognitive task performance measures across many species found moderate support for both temporal (same task presented at different times) and contextual (different tasks that are designed to quantify the same cognitive trait) short-term repeatability of cognitive performance [19], suggesting the use of psychometric tasks is a valid tool for the study of cognition. However, the long-term repeatability of cognitive performance is largely unknown. This has a number of important implications—first, if individual cognitive performance is stable over time, this indicates the existence of a cognitive phenotype on which selection might act. Accordingly, an understanding of the long-term repeatability of cognitive performance, coupled with studies investigating the causes and consequences of individual variation in cognitive performance [6,12,23,24], has the potential to further our understanding of the factors governing cognitive evolution. Second, quantifying the long-term repeatability of cognitive performance can be used to test predictions about developmental influences on cognitive performance. Over long time periods the social and non-social environment of an individual is likely to change. Quantifying the long-term repeatability of cognitive performance during such time frames can be used to determine if these changes are related to subsequent changes in cognitive performance. Finally, a deeper understanding of how stable cognitive traits are over long timeframes will shed light on the replicability of experimental findings.

Previous work on the Western Australian magpie *Cracticus tibicen dorsalis* has shown cognitive performance is positively associated with group size and is repeatable over a two-week time-frame [6]. The same study also identified positive associations in performance across four different cognitive tasks, pointing towards a general cognitive factor, or general cognitive performance (commonly referred to as *g* in the human literature [25]). In this study we investigated the long-term repeatability of cognitive performance (by task) and general cognitive performance (*g*) in the Western Australian magpie. To do this, we quantified cognitive performance in four well-studied, ecologically relevant traits [26–28]; inhibitory control, associative learning, reversal learning and spatial memory. Inhibitory control, the ability to inhibit prepotent responses, has been implicated in adaptive decision-making [29–31]. Associative learning enables the acquisition of predictive contingencies between cues in the environment, and reversal-learning enables the flexible readjustment of learnt predictive contingencies [26,27,32]. Spatial memory is likely to be important in remembering locations of resources and territory boundaries [33]. These traits were quantified in the same individuals twice; once in 2015 (reported in Ashton *et al.* 2018 [6]) and once in 2018, using causally identical but visually distinct versions of the same task, to control for the potentially confounding effect of memory on performance. Although group sizes had been stable at our study site for 5 years [6], over the course of the 2018 breeding season there were a number of group splits, whereby some of the larger groups underwent a single split into two smaller groups. Given the positive relationship between group size and cognitive performance in this species [6], one might predict a change in group size could cause a corresponding change in cognitive performance. Therefore, we also investigated if changes in group size account for a significant amount of variation in the repeatability of cognitive performance.

## 2. Methods

### 2.1. Study site and population

This study was conducted on a population of Western Australian magpies in the Guildford and Crawley suburbs of Perth, Western Australia. The Western Australian magpie is a large (250–400 g) cooperatively breeding bird occurring in territorial groups of 3–12 adults [34]. The study population is comprised of an average of 86 individuals (range 70–122) from up to 18 groups annually, of which the majority are ringed, allowing individual identification and the presentation of cognitive tasks [6,35–37]. Thirty-four individuals were tested in both 2015 and 2018; 32 on the inhibitory control task, 29 on the associative learning and reversal learning task, and 30 on the spatial memory task. 27 individuals completed the entire cognitive test battery at both testing points. All individuals tested were adults (individuals greater than 3 years old, *N* = 17 females, *N* = 17 males).

### 2.2. Cognitive performance

**Figure 1.**
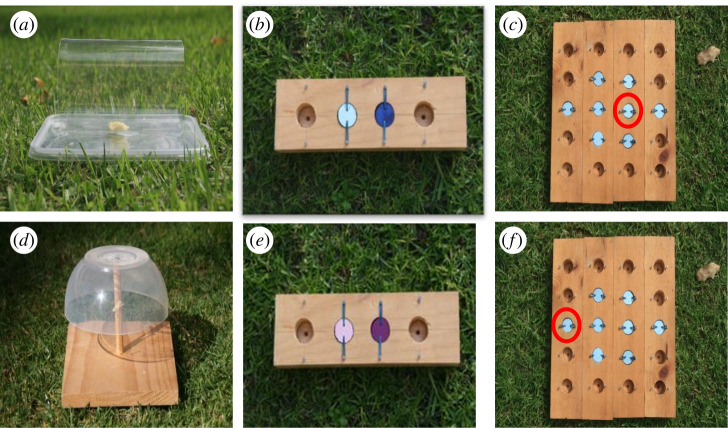
The cognitive tasks used to quantify inhibitory control (*a*,*d*), associative learning (*b*,*e*), reversal learning (*b*,*e*), and spatial memory (*c*,*f*). Tasks *a*-*c* represent the cognitive test battery used in 2015 and tasks (*d*–*f*) represent the cognitive test battery used in 2018.

To quantify inhibitory control, individuals were presented with two detour-reaching tasks (figure 1*a*,*d*). Detour-reaching tasks quantify inhibitory control by testing an individual's ability to inhibit a prepotent response [38]. In the two tests used, individuals were required to inhibit pecking at a transparent surface, and detour around it in order to gain access to a food reward. In 2015, individuals were presented with a transparent open-ended cylinder—test subjects had to detour around the transparent cylinder in order to retrieve the food reward from the open end (figure 1*a*). In 2018, individuals were presented with a transparent ‘umbrella’—for this task the food reward could be accessed by detouring underneath the transparent dome (figure 1*d*). For both the 2015 and 2018 task, once an individual successfully detoured without pecking the transparent surface three times in a row it was considered to have passed the inhibitory control task. The number of trials taken to pass was the measure of success. There was at least a one-minute interval between trials, with a maximum of 10 trials. Individuals that did not pass the task within 10 trials were assigned a score of 10. Where possible, all trials on a focal bird were carried out in a single day (cognitive testing did not exceed three days for any individuals). The two inhibitory control tasks were designed to be of comparable difficulty—it was reasoned that inhibiting the pecking response and either (i) detouring around to the open ends of a cylinder or (ii) underneath a transparent dome, would require comparable levels of inhibitory control. Individuals, on average, took a similar number of trials to complete each of the tasks (see Results section), suggesting they were indeed of similar difficulty.

To quantify associative learning individuals completed a colour association task, whereby they were presented with a foraging grid containing two wells covered with lids of two different shades of the same colour (figure 1*b*,*e*). For each test subject one shade of colour was randomly chosen to be the rewarded colour for the duration of the experiment, such that when the lid was pecked, the bird would gain access to a food reward (a small piece of grated cheese). Birds were trained to peck the lids and retrieve food from the wells in three sequential steps—first, the task was presented with no lids covering the wells, second, with lids partially covering the wells, and third, with lids fully covering the wells. Lid colour in the training phase was yellow, a colour not used in any of the subsequent experimental trials. Once a bird had successfully searched the wells when fully covered by lids three times in a row, it moved onto the experimental trials of the associative learning task. In 2015 the colours used were dark-blue and light-blue (figure 1*b*), in 2018 the colours used were dark-purple and light-purple (figure 1*e*). Shades, rather than distinct colours, were used to reduce the chances of pre-existing biases for particular colours influencing performance. On the first experimental trial individuals were allowed to search both wells to demonstrate that only one was rewarded, but in all subsequent trials, the array was removed after the first well had been chosen, to ensure there was a cost associated with incorrect choices (i.e. the bird not getting the food reward). Test subjects had a minute to complete an experimental trial, there was a minimum of a one-minute interval between trials, and a maximum of 50 trials per focal individual per day (range of number of trials per day = 10–50, average number of trials per day = 18.25). If the maximum number of trials was reached on one day, trials were continued the following day. The position of the baited well (left or right) was randomized between trials to ensure that colour was the cue being associated with the food reward, not location. To control for olfactory cues both wells were wiped with cheese before trials. Individuals were considered to have passed the task when they chose the rewarded well in 10 out of 12 consecutive trials (this represents a significant deviation from binomial probability; binomial test: *p* = 0.039). The number of trials taken to reach this criterion was the associative learning score.

To quantify reversal learning the exact same protocol as associative learning was carried out 24 h after the completion of the associative learning task, except that the previously unrewarded colour was now the rewarded colour. The measure of cognitive performance was also the same as in the associative learning task. The number of trials per day ranged between 11 and 50, and the average number of trials per day was 22 for the reversal learning task. While the associative and reversal learning tasks are not entirely independent, there are a number of studies suggesting that performance on these two tasks may be dissociated or even inversely related [39,40]. There is also a large amount of literature providing evidence that reversal learning relies on different molecular/neural mechanisms compared to associative learning, and involves different brain regions [41,42]. For these reasons, the majority of literature presents associative learning and reversal learning as distinct traits (see Cauchoix *et al.* [19] and references therein for an overview).

To quantify spatial memory, individuals were presented with a foraging grid containing eight wells covered with light-blue lids like those used in both the associative and reversal learning tasks (figure 1*c*,*f*) [28]. The wells were arranged equidistantly apart in three rows (two wells on the first row, four wells on the second row, two wells on the third row). One well was randomly chosen to be the rewarded location for the duration of the experiment. The spatial memory experiment consisted of five presentations. First, individuals were presented with the array in a ‘baseline’ trial, whereby they were allowed to search the grid for the hidden food reward. Five minutes later individuals were presented with the grid a second time in a ‘memory’ trial. 24 h and 48 h after the memory trial, individuals were presented with the grid again. The cumulative number of wells searched before finding the rewarded well in the 24 h and 48 h presentations were the spatial memory score. Individuals were presented with the grid a fifth time 5 min after the 48 h trial in an olfactory trial—this time the grid was unbaited and rotated 180 degrees. The foraging grid would appear identical to the test subjects, but the position of the previously baited well would be on the opposite side of the grid compared to the other phases of the experiment. If individuals were relying on olfactory cues to locate the food reward, we predict that they would search the previously rewarded well (now in a different location, unbaited). If individuals were relying on spatial memory, we predict that individuals would search the well opposite the previously baited well. The number of wells searched in the fifth trial did not contribute to the spatial memory score. Analyses on the 2015 and 2018 datasets (paired *t*-tests comparing the number of wells searched in the 48 h post-training phase trial and the fifth trial) confirmed that individuals chose wells based on location, not olfactory cue, in the fifth trial [6]—i.e. individuals continued to choose the now unbaited well in the fifth trial (2015 paired *t*-test: *t* = 1.069, *p* = 0.294; 2018 paired *t*-test, *t* = 1.283, *p* = 0.213).

To control for the potentially confounding effects of social learning and social interference, tasks were presented to test subjects when they were greater than 10 m away from any other individual. This is possible as magpies often forage greater than 10 m away from each other [36]. Experimenters baited the tasks out of sight from test subjects. Tasks were then placed on the ground 5 m in front of the focal individual before the experimenter moved 5 m away to allow the test subject to interact with the task. Trials were discontinued if another magpie approached the test subject. ‘Test order’ had been included as an explanatory term in previous analyses on the 2015 dataset to verify that social learning did not influence performance—if social learning was influencing performance we predicted that individuals tested later in the group would perform better; this was not the case [6]. Trials were carried out early in the morning (5 am-10 am) to reduce the chance of satiation influencing task participation, and were recorded live by observers.

### 2.3. Statistical analyses

Previously, a principal components analysis (PCA) on the 2015 dataset found individual performance was highly correlated across the four tasks, indicative of a general intelligence factor [6] (hereafter referred to as ‘general cognitive performance’ (GCP)). A PCA was also carried out on the 2018 dataset. Only principal components with an eigenvalue greater than 1 were extracted from the PCA. GCP is generally accepted to exist when performance in all tasks positively loads onto the first principal component and accounts for greater than 30% of total task variance [43]. In addition to performance on the four cognitive tasks, the repeatability of GCP was also investigated.

Repeatability analyses were carried out in R (v. 4.1.0, http://www.r-project.org) using the rptR package [44]. We checked that all models met assumptions (homogeneity, normality of residuals) using the DHARMa package in R [45]. Separate generalized linear mixed model repeatability estimates with Poisson distribution were used to determine the repeatability of cognitive performance on each of the four cognitive tasks. A linear mixed model repeatability estimate with a Gaussian distribution was used to determine the repeatability of GCP. To investigate the effect of changes in group size on the repeatability of cognitive performance, ‘change in group size’—the difference in group size between 2015 and 2018—was included as a fixed effect in models for all tasks. Comparison of adjusted repeatability estimates, accounting for changes in group size, against unadjusted repeatability estimates, allowed us to determine if changes in social structure were sources of variation in repeatability estimates of cognitive performance.

## 3. Results

In 2015, the time taken to pass the inhibitory control task ranged between 3 and 10 trials (mean number of trials = 6.28 ± 0.469). In 2018, the time taken to pass the inhibitory control task ranged between 3 and 10 trials (mean number of trials = 5.91 ± 0.536). On average it took individuals 0.37 (±0.573) fewer trials to pass the inhibitory control task in 2018 compared to 2015 (the change in the number of trials taken to complete the inhibitory control tasks ranged between −7 ± 6). In 2015, the time taken to pass the associative learning task ranged between 10 and 65 trials (mean number of trials = 24.38 ± 2.843). In 2018, the time taken to pass the associative learning task ranged between 10 and 48 trials (mean number of trials = 19.76 ± 2.022). On average it took individuals 4.62 (±2.144) fewer trials to pass the associative learning task in 2018 compared to 2015 (the change in the number of trials taken to complete the associative learning tasks ranged between −46 and +19). In 2015, the time taken to pass the reversal-learning task ranged between 11 and 94 trials (mean number of trials = 33.9 ± 4.432). In 2018, the time taken to pass the reversal-learning task ranged between 11 and 73 trials (mean number of trials = 21.86 ± 2.564). On average it took individuals 12.03 (±2.949) fewer trials to pass the reversal-learning task in 2018 compared to 2015 (the change in the number of trials taken to complete the reversal learning tasks ranged between −44 and +6). In 2015, the time taken to pass the spatial memory task ranged between 2 and 27 trials (mean number of trials = 8.53 ± 0.987). In 2018, the time taken to pass the spatial memory task ranged between 3 and 15 trials (mean number of trials = 6.57 ± 0.558). On average it took individuals 1.97 (±0.812) fewer searches to complete the spatial memory task in 2018 compared to 2015 (the change in the number of searches taken to complete the spatial memory task ranged between −15 and +3). Similar to results obtained during cognitive testing in 2015 [6] (table 1), we found evidence for general cognitive performances in 2018 (table 1), whereby individuals that performed well on one task also tended to perform well in the other tasks. The first principal component extracted with an eigenvalue over one accounted for 70.65% of total variance in task performance (compared to 64.56% in 2015). In 2015, general cognitive performance scores ranged between −2.843–0.949 (mean = −0.215 ± 0.203). In 2018, general cognitive performance scores ranged between −2.988 and 1.112 (mean = 0.001 ± 0.192). On average general cognitive performance scores were 0.215 (±0.142) higher in 2018 compared to 2015 (the change in general cognitive performance scores ranged between −1.012 and +1.941).

**Table 1.** Principal components analysis for performance on the cognitive test battery in 2015 and 2018.

2015		2018	
Task	PC1	task	PC1
inhibitory control	0.703	inhibitory control	0.364
associative learning	0.789	associative learning	0.824
reversal learning	0.870	reversal learning	0.787
spatial memory	0.841	spatial memory	0.851
Eigenvalue	2.582	eigenvalue	2.826
% of total variance explained	64.56	% of total variance explained	70.65

**Figure 2.**
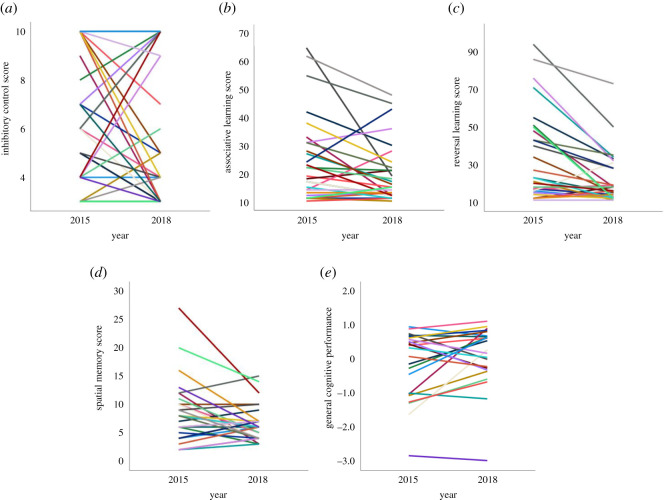
Repeatability of cognitive performance in an (*a*) inhibitory control task (*N* = 32 individuals), (*b*) associative learning task (*N* = 29 individuals), (*c*) reversal-learning task (*N* = 29 individuals), (*d*) spatial memory task (*N* = 30 individuals), and (*e*) general cognitive performance (*N* = 27 individuals), across a three-year time period.

We found significantly high levels of long-term repeatability in two out of four measures of cognitive performance (table 2 and figure 2). Associative learning (*N* = 29 individuals) and reversal learning (*N* = 29 individuals) were significantly repeatable, but spatial memory (*N* = 30 individuals) and inhibitory control (*N* = 32 individuals), although trending towards significance, were not (table 2). General cognitive performance (*N* = 27 individuals) was also significantly repeatable (table 2). As predicted, long-term repeatability estimates were lower than short-term repeatability estimates (table 2). Comparison of unadjusted and adjusted (accounting for changes in group size) long-term repeatability estimates show changes in group size did not account for a significant amount of variation in the long-term repeatability of cognitive performance (i.e. adjusted repeatability estimates remained either significant or not significant after considering group size effects, table 2).

**Table 2.** Unadjusted and adjusted (for changes in group size) repeatability estimates for performance in four cognitive tasks and general cognitive performance (GCP). Unadjusted short-term repeatability estimates reproduced from Ashton *et al*. [6] Significant repeatability estimates are in bold.

cognitive test	type of R	*R*	SE	*N*	confidence intervals
inhibitory control	unadjusted short-term *R*	**0.806**	0.049	56	0.691, 0.882
	unadjusted long-term *R*	0.282	0.132	32	0, 0.464
	long-term R adjusted for change in group size	0.301	0.135	32	0, 0.468
associative learning	unadjusted short-term R	**0.970**	0.010	46	0.946, 0.983
	unadjusted long-term R	**0.599**	0.133	29	0.277, 0.796
	long-term R adjusted for change in group size	**0.583**	0.140	29	0.274, 0.804
reversal learning	unadjusted short-term R	**0.975**	0.008	46	0.954, 0.986
	unadjusted long-term R	**0.485**	0.149	29	0.164, 0.720
	long-term R adjusted for change in group size	**0.466**	0.157	29	0.110, 0.730
spatial memory	unadjusted short-term R	**0.932**	0.021	46	0.879, 0.963
	unadjusted long-term R	0.401	0.159	30	0, 0.609
	long-term R adjusted for change in group size	0.319	0.163	30	0, 0.587
GCP	unadjusted short-term R	**0.976**	0.070	43	0.959, 0.987
	unadjusted long-term R	**0.708**	0.113	27	0.433, 0.869
	long-term R adjusted for change in group size	**0.673**	0.119	27	0.391, 0.846

## 4. Discussion

Until now, the vast majority of studies investigating the repeatability of cognitive performance have focused on short-term repeatability [19]. While these studies are crucial, it is vital that the long-term repeatability of cognitive performance is known as well. We identified significant long-term repeatability of cognitive performance in associative learning and reversal learning tasks, and a non-significant trend in long-term repeatability of cognitive performance in inhibitory control and spatial memory tasks. Measures of general cognitive performance, derived through PCA analyses, also showed significant long-term repeatability. There was a trend for individuals to perform better in the 2018 cognitive test battery compared to the 2015 cognitive test battery, suggesting individual performance may improve with repeated testing. However, in a separate study, magpies tested on three causally identical but visually distinct inhibitory control tasks showed no improvement in task performance over time, indicating repeated testing does not confound cognitive performance [46]. Higher long-term repeatability estimates were generally observed in tasks where among-individual variance in task performance was higher. This reiterates recent suggestions that repeatability estimates should be carefully interpreted alongside measures of variance [47]. Although we report significant long-term repeatability in GCP, it is worth noting that our measures of GCP are derived from a comparatively small cognitive test battery (studies in human psychometric testing often include upwards of 10 tests [25,48]) covering a relatively narrow range of cognitive traits. It is therefore possible that our measures of GCP are the result of methodological construct, and should be interpreted with caution [48].

Long-term repeatability estimates of cognitive performance can be used to determine the stability of cognitive traits, which in turn can be used to identify the existence of cognitive phenotypes on which selection might act. In this vein, a number of recent studies have reported positive relationships between cognitive performance and proxies of fitness [11–13,40,49]—these results have been used to conclude that directional selection on cognition may be operating in these species. However, in studies examining the relationship between cognitive performance and fitness, the long-term stability of cognitive traits is often unknown and cognitive performance and measures of fitness are often recorded at different time points [12], making such conclusions problematic. The long-term repeatability estimates we report here may go some way to validating these conclusions, and therefore have important implications for our understanding of how selection may act on cognitive traits (although it is necessary to determine the long-term repeatability of cognitive performance in all study species where conclusions about cognitive performance and selection are made). Crucially, in order to fully understand how selection acts on cognitive traits it is also necessary to understand the genetic basis of individual variation in cognition [50,51].

Quantifying the long-term repeatability of cognitive performance also allows the developmental and environmental influences on cognitive performance to be explored. Here, we investigated whether changes in the social environment account for a significant proportion of variation in the repeatability of cognitive performance. This might be predicted as a relationship between group size and cognitive performance has been found in Australian magpies previously [6]. The study population experienced changes in group size between the time cognitive performance was first quantified in 2015 and last quantified in 2018—this afforded us the opportunity to determine if changes in the social environment cause corresponding changes in cognitive performance. We found no evidence that changes in group size influenced the repeatability of cognitive performance. This may be for a number of reasons; it is possible that changes in the social environment need to occur over a longer period of time in order to induce cognitive changes. Alternatively, whereas all the individuals tested in the current study were adults, it may be that the social environment during early life is particularly important in shaping cognitive phenotypes [6,52]. Indeed, our previous work shows that group size-related differences in Australian magpies emerge over the first 300 days post-fledging [6]. Coupled with the long-term repeatability estimates reported here, this suggests that the social environment during early life is key to cognitive development.

Long-term repeatability estimates of cognitive performance can help address the replication crisis by determining the replicability of results. Although long-term repeatability estimates were lower than short-term repeatability estimates, these results indicate that measures of cognitive performance in Australian magpies are stable in the long-term in at least some cognitive traits. Assuming that all potential explanatory variables are kept constant, this suggests that experimental findings should be replicable in this study species [37]. Notably, repeatability of performance in the inhibitory control task was the lowest—previous work has indicated that detour-reaching tasks are particularly susceptible to non-cognitive factors and confounding variables [53,54], which may have contributed to this.

In summary, the findings from this study demonstrate how investigation into the long-term repeatability of cognitive performance can reveal insights into the stability of cognitive phenotypes, the findings of which have important implications for the study of cognitive evolution.

## Data Availability

The data are provided in electronic supplementary material [55].
